# A new species of *Eriotheca* (Malvaceae, Bombacoideae) from coastal areas in northeastern Brazil

**DOI:** 10.3897/phytokeys.167.57840

**Published:** 2020-11-20

**Authors:** Jefferson Carvalho-Sobrinho, Aline C. da Mota, Laurence J. Dorr

**Affiliations:** 1 Universidade Federal do Vale do São Francisco – UNIVASF, Colegiado de Ciências Biológicas, Petrolina, Pernambuco, 56300-990, Brazil Universidade Federal do Vale do São Francisco Petrolina Brazil; 2 Universidade Federal Rural de Pernambuco – UFRPE, Departamento de Ciências Florestais, Recife, Pernambuco, 52171-900, Brazil Universidade Federal Rural de Pernambuco Recife Brazil; 3 Universidade de Pernambuco – UPE, Instituto de Ciências Biológicas, Recife, Pernambuco, 50100-130, Brazil Universidade de Pernambuco Recife Brazil; 4 Department of Botany, MRC-166, Smithsonian Institution, P.O. Box 37012, Washington, D.C. 20013-7012, USA Smithsonian Institution Washington DC United States of America

**Keywords:** ‘Bombacaceae’, ‘embiruçú’, endemism, plant taxonomy, restinga, sandy soils

## Abstract

A new species of *Eriotheca* (Malvaceae, Bombacoideae) from coastal areas in the northeastern Brazilian states of Alagoas and Bahia is described and illustrated. *Eriothecaalversonii* inhabits Atlantic coastal forest and is found principally on sandy soils in restinga vegetation. It is most similar morphologically to *E.parvifolia*. Both species have 3-foliolate leaves and short petioles on fertile branches, but the new species has smaller flowers, truncate to crenulate calyces, and smaller globose to subglobose capsules. The affinities of *E.alversonii* to morphologically similar species and its phenology are discussed. A distribution map and preliminary assessment of its conservation status are provided.

## Introduction

*Eriotheca* Schott & Endl. is one of 17 genera in the Bombacoideae (Malvaceae), a pantropical subfamily that includes ca. 160 species (Carvalho-Sobrinho et al. 2016). *Eriotheca* is restricted to South America (Robyns 1963, 1968, 1979; Robyns and Nilsson 1975, 1981; Fernández-Alonso 1999, 2003) and where it occurs, it is an important element in the physiognomy and community structure of both seasonally dry tropical and moist forests (e.g., Macbride 1956; Ferreyra 1986; Linares-Palomino and Ponce Alvarez 2005; Thomas et al. 2008; Pennington et al. 2009). It includes ca. 25 species of which 19 occur in Brazil mainly in cerrado and Atlantic coastal forest (Duarte and Esteves 2011; Carvalho-Sobrinho 2013; Carvalho-Sobrinho et al. 2015; Macedo et al. 2018).

Molecular phylogenetic analyses place *Eriotheca* and *Pachira* Aubl. in a clade characterized by striate seeds (Duarte et al. 2011; Carvalho-Sobrinho et al. 2016), alternate eophylls, lack of prickles on trunks and branches, leaflets with brochidodromous venation (Carvalho-Sobrinho et al. 2016), and only two rows of ovules in the ovary (Franca et al. 2018). These analyses, however, also suggest that *Eriotheca* and *Pachira* as currently circumscribed are not monophyletic, and further molecular and taxonomic sampling is necessary to resolve the relationships between these two genera (Carvalho-Sobrinho et al. 2016). Until such sampling is completed, it would be premature to place *Eriotheca* in synonymy with *Pachira* and as a consequence new species have been described in both genera while maintaining their traditional circumscriptions (Duarte and Esteves 2011; Carvalho-Sobrinho 2013; Carvalho-Sobrinho et al. 2014b, 2015; Macedo et al. 2018).

*Eriotheca* differs in a number of characters from *Pachira*. In addition to the smaller flowers in *Eriotheca* (up to 55 mm long), it also has filaments freely originating from a staminal tube (phalanges are absent), a single whorl in the androecium (Robyns 1963; Carvalho-Sobrinho 2013; Carvalho-Sobrinho et al. 2015), often unilaterally apiculate petals (Robyns 1963), reniform anthers (Carvalho-Sobrinho 2013), a different indumentum on the external surface of the petals, and a glabrous tube (Carvalho-Sobrinho, pers. obs.).

*Eriotheca* is characterized by mostly medium to emergent trees, leaves that are palmately compound with leaflets articulate at the petiole apex, flowers with a persistent calyx that is accrescent in fruit, a receptacle often with external nectaries, an androecium with 18 to 170 stamens and dorsifixed anthers, capsules with copious brown kapok, and numerous, striate seeds usually up to 1 cm in diameter (Robyns 1963). An underground xylopodium-like structure was reported for *E.saxicola* Carv.-Sobr. (Carvalho-Sobrinho 2013). *Eriotheca* flowers are pollinated by bees, bats or hawkmoths (Oliveira et al. 1992; Sazima et al. 1999; MacFarlane et al. 2003).

The taxonomy of *Eriotheca* is challenging because type specimens are often phenologically incomplete (cf. Robyns 1963) and affect species circumscriptions because it is difficult to match leaves, flowers, and fruit characters from different specimens (Carvalho-Sobrinho and Queiroz 2008, 2010; Carvalho-Sobrinho et al. 2013a, b, 2014a, b). Identification of *Eriotheca* specimens traditionally has relied largely on floral characters, especially the length of pedicels, flower bud shape, calyx shape and indumentum, petal shape, and staminal tube shape (Robyns 1963). More recently, micromorphological characters from leaves have been used to circumscribe and diagnose species (Duarte and Esteves 2011; Carvalho-Sobrinho et al. 2015) and to elaborate identification keys (Duarte and Esteves 2011), although this innovation has limited applicability in fieldwork or herbarium research.

Ongoing studies on the systematics of Neotropical Bombacoideae (Carvalho-Sobrinho and Queiroz 2008, 2010, 2011; Carvalho-Sobrinho et al. 2009, 2012, 2013a, b, 2014a, b, 2015, 2016; Carvalho-Sobrinho and Dorr 2017) have revealed herbarium specimens of *Eriotheca* from the coastal Atlantic states of Alagoas and Bahia in northeastern Brazil that are noteworthy because their leaves and fruits are smaller than others in the genus. Careful study of these specimens has led to the recognition of a new species, which is described and illustrated here. Notes on this species’ distribution and phenology, comments on morphologically similar species, and a preliminary assessment of its conservation status are provided.

## Material and methods

This study was based on examination of herbarium collections, field observations, and digital images of specimens. Specimens were studied by visits to or loans from the following herbaria: ALCB, ASE, CEPEC, F, HUEFS, K, MBM, MO, NY, RB, SP, SPF, and US. Images of additional herbarium specimens were studied through the following websites: JSTOR Global Plants (https://plants.jstor.org/) and INCT – Herbário Virtual da Flora e dos Fungos (http://inct.splink.org.br/). Descriptions and measurements are based on dry herbarium specimens. The distribution map was prepared using QGIS v.3.12.2 (QGIS Development Team 2020). A preliminary extinction risk assessment of the new species was made using the IUCN Red List Categories and Criteria (IUCN 2019). Georeferenced specimen data were imported into GeoCAT (Bachman et al. 2011) to estimate the extent of occurrence (EOO) and the area of occupancy (AOO) using 2 × 2 km grid cells.

## Taxonomic treatment

### Eriotheca alversonii

Taxon classificationPlantaeMalvalesMalvaceae

Carv.-Sobr. & Dorr sp. nov.

40129775-535E-5848-A25B-A6D7468429F9

urn:lsid:ipni.org:names:77212952-1

Figs 1 , 2

#### Diagnosis.

Similar to *Eriothecaparvifolia* (Mart.) A.Robyns in its 3-foliolate, glabrous leaves, and short petioles on fertile branches, but differing in its linear-oblong (vs. large elliptic) flower buds, smaller (3–4 × 3–5 vs. 7 × 8–11 mm) cupuliform (vs. campanulate) calyces with apices truncate to crenulate (vs. mostly 3–5-lobed), fewer stamens (ca. 70 vs. ca. 120), and smaller capsules (15–21 vs. 30–35 mm long).

#### Type.

Brazil. Bahia: Maraú, entrada à direita ca. 3 km da entrada da cidade, propriedade particular ‘Espaço 21’, 14°10'27"S, 38°59'53"W, 7 m a.s.l., 08 Jul 2011 (lf, fl buds, fl), *J.G. Carvalho-Sobrinho et al. 3126* (holotype: HUEFS).

#### Description.

Treelets or more often trees to 20 m tall; trunks to 50 cm dbh; buttresses 40 × 60 cm; branches often blackish in herbarium specimens. Terminal buds often persistent at branch apices, 5–11 mm long, attenuate and falcate apically. Leaves palmately compound; petioles on fertile branches up to 8 mm long (to 20 mm long on vegetative branches); petiolules absent to greatly reduced; leaflets 1–3(–5, in vegetative branches), 15–46(107) × 8–56 mm, coriaceous; proximal leaflets 8–27 mm wide; distal leaflets 8–56 mm wide; leaflet length-to-width ratio (1.5)1.9–2.5(3); leaflets narrowly obovate, elliptic to widely elliptic in fertile branches, rarely obcordate, apices retuse to emarginate, bases cuneate, margin entire, revolute, strongly revolute at base, glabrous on both surfaces, except for sparse microtrichomes on abaxial surface, discolorous, adaxial surface of fresh leaflets dark green and abaxial surface light green, abaxial surface of dry leaflets often reddish-brown, midrib prominent abaxially, secondary veins 7–10, impressed on both surfaces, intersecondary veins impressed on abaxial surface. Inflorescences axillary, 1–6-flowered cymes, borne on younger, terminal often leafy branches; pedicels 10–22 mm long, covered with blackish indumentum; bracteoles caducous. Flowers linear-oblong in bud, ca. 25 mm long; receptacles lacking glands; calyces 3–4 × 3–5 mm, cupuliform, truncate to crenulate, accrescent in fruit, outer surface covered with ferruginous indumentum, blackish when very young; petals 15–23 × 3–6 mm, oblanceolate, unilaterally apiculate, tomentose on both faces, internally with longitudinal lines of longer trichomes (sericeous) on one longitudinal half, whitish when fresh; stamens ca. 70, cream-colored when fresh; staminal tube 5 mm long, oblong, slightly expanded at apex, producing free filaments 11 mm long; ovary subglobose, the style inconspicuously 5–lobed. Capsules 15–21 × 13–20 mm, globose to subglobose, externally glabrous, kapok abundant, brown. Seeds numerous, 5 mm in diam., pyriform, glabrous.

**Figure 1.**
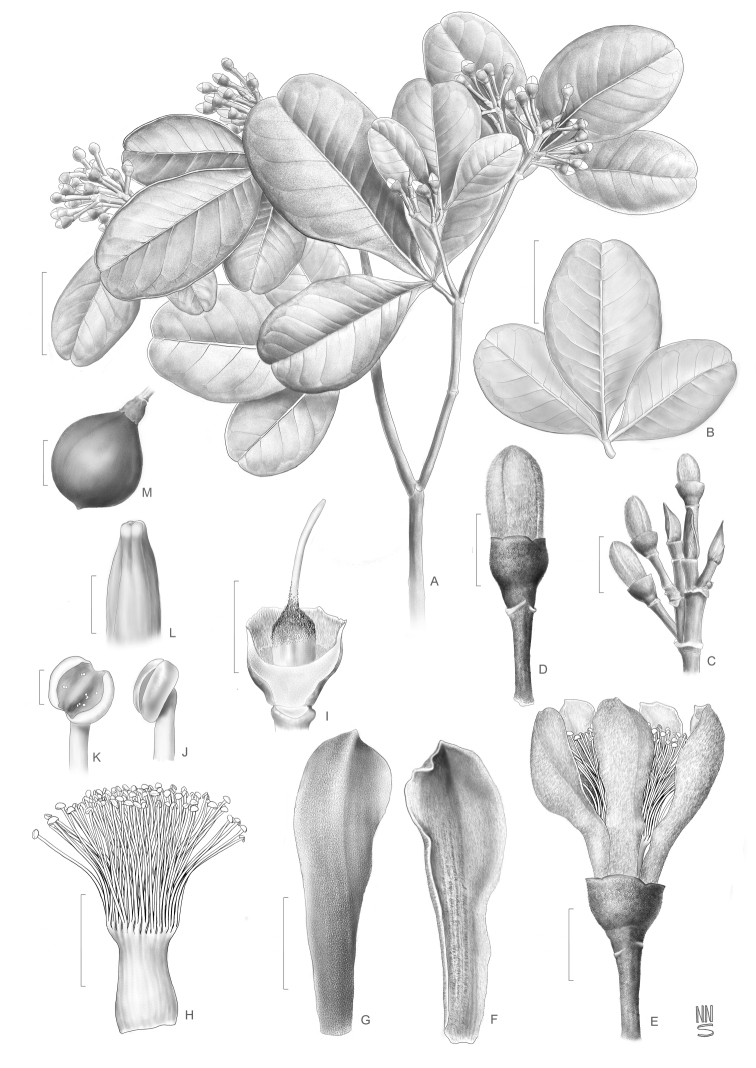
*Eriothecaalversonii***A** flowering branch **B** three-foliolate leaf **C** flower buds and vegetative terminal buds **D** flower bud **E** flower **F, G** petals; adaxial and abaxial views **H** staminal tube **I** ovary **J, K** anthers; undehisced and dehisced **L** stigma **M** fruit. All drawn from the holotype, except for fruit (*L.A. Mattos Silva 1769*). Scale bars: 3 cm (**A, B**); 1 cm (**C, M**); 5 mm (**D–I**); 0.5 mm (**J–L**).

#### Phenology.

Flower buds in June and July, open flowers in August and September and mature fruits in October and December to February.

#### Distribution and habitat.

*Eriothecaalversonii* is known from coastal vegetation mainly over quaternary white sand (restinga forest) or less frequently on clay-sandy soils in transitional vegetation between restinga forest and wet dense forest (“floresta ombrófila densa”), in the northeastern states of Alagoas and Bahia, Brazil.

#### Conservation status.

*Eriothecaalversonii* is known from 19 collections from six different localities (municipalities). The extent of occurrence (EOO) of this species has been calculated to be 18,466 km^2^, which qualifies the species for the Vulnerable (VU) category, and the area of occupancy (AOO) was estimated to be 28 km^2^, which qualifies it for the Endangered (EN) category (Bachman et al. 2011; IUCN 2019). Based on herbarium specimen labels, three collections of *E.alversonii* were made inside one state-level protected Reserve (APA de Santa Rita) as explicitly stated in collectors’ descriptions, and an additional four collections probably were made inside state- (APA Pratigi and APA Marituba do Peixe) or federal-level (Reserva Extrativista de Canavieiras) protected areas; nevertheless, all these protected areas allow sustainable use of natural resources and none of them are of the highest level of protection (level I or II) described by the IUCN (Dudley 2008). Furthermore, restinga habitat currently is being lost at an accelerated rate due to anthropogenic pressures (Rocha et al. 2007; Pergentino and Landim 2014) and most collections of *E.alversonii* were made on farms. Therefore, due to the rapid rate of deforestation of the much fragmented restinga vegetation and the small AOO (32 km^2^) of *E.alversonii*, we consider this species to be Endangered (EN category) according to IUCN criteria (IUCN 2019).

#### Etymology.

The specific epithet honors the North American botanist Dr. William (‘Bil’) Surprison Alverson (b. 1953) who has contributed greatly to our understanding of the phylogeny and systematics of Neotropical Bombacoideae.

#### Additional specimens examined.

Brazil. **Alagoas**: Barra de São Miguel, 9°50'25"S, 35°54'28"W, 28 Aug 1981 (lf, buds), *M.N.R. Staviski et al. 940* (MAC); *ibidem*, loteamento próximo ao Rio Niquim, 24 Jan 2008 (lf, fr), *L. Omena 4* (MAC); Marechal Deodoro, APA de Santa Rita, Sítio Campo Grande, vegetação sobre cordões litorâneos, 25 Sept 1990 (lf, fl, fr), 10°11'4"S, 36°29'50"W, *R.P. Lyra-Lemos 1750* (ALCB, MAC, SP); *ibidem*, APA de Santa Rita, Sítio Campo Grande, 25 Sept 1990 (lf, fl), *R.P. Lyra-Lemos & J.E. de Paula 1762* (MAC, SPF); *ibidem*, APA de Santa Rita, próximo a Campo Grande, 10°11'4"S, 36°29'50"W, 24 Aug 1999 (lf, fl), *R.P. Lyra-Lemos & I.A. Bayma 4207* (ESA, MAC, SP); *ibidem*, Dunas do Cavalo Russo, 04 Feb 2009 (lf), *Chagas-Mota & L.M. Leão 1826* (MAC); *ibidem*, Dunas do Cavalo Russo, 12 Feb 2009 (lf), *Chagas-Mota 1987* (MAC); *ibidem*, Dunas do Cavalo Russo, 9°42'37"S, 35°53'42"W, s.d. (st), *J.C. Lemos 28* (MAC); *ibidem*, Dunas do Cavalo Russo, Povoado Cabreiras, 30 Aug 2008 (lf, fl), *R.P. Lyra-Lemos et al. 11457* (MAC); *ibidem*, encosta de tabuleiro próximo às dunas do Cavalo Russo, 09 Dec 1998 (lf, fr), *R.P. Lyra-Lemos 4086* (MAC, SP); *ibidem*, Mucuri, próximo a Campo Grande, vegetação sobre cordões arenosos, 24 Aug 1999 (lf, fl), *R.P. Lyra-Lemos & I.A. Bayma 4235* (ASE, MAC); *ibidem*, próximo Praia do Francês, 31 Jan 1982 (fr), *D. Araújo s.n.* (RB1382616); *ibidem*, Sítio Bom Retiro, 09°41'52"S, 35°53'36"W, 07 Feb 2007 (lf, fr), *A.I.L. Pinheiro & S. Mendes 327* (MAC). Penedo, Marituba do Peixe, 19 Aug 2006, *M.N. Rodrigues et al. 1983* (lf, fl), 10°17'55"S, 36°25'37"W (MAC). **Bahia**: Cairu, Gamboa, 13 Aug 1993 (lf, imm fr), *M.L. Guedes et al. s.n.* (ALCB 26059); *ibidem*, Fazenda Bela Vista, 14 Sept 1993 (lf, imm fr), *M.L. Guedes et al. s.n.* (RB 426439); Maraú, estrada à direita para uma propriedade particular, a ca. 3 km da entrada da cidade de Maraú, 14°09'32"S, 39°00'1"W, 19 Aug 2008 (lf, fl), *L.P. Queiroz et al. 13018* (HUEFS); *ibidem*, entrada à direita ca. 3 km da entrada da cidade, propriedade particular ‘Espaço 21’, 14°10'17"S, 38°53'53"W, 20 m a.s.l., 07 Jul 2011 (lf, buds), *J.G. Carvalho-Sobrinho et al. 3125* (HUEFS); Nilo Peçanha, ramal para o povoado de Itiuca, ramal com entrada no km 8 da rodovia Nilo Peçanha/Cairu (BA 250), lado direito, piaçaval em capoeira, solo arenoso, 24 Oct 1984 (lf, fr), *L.A. Mattos Silva & T.S. Santos 1769* (CEPEC).

**Figure 2.**
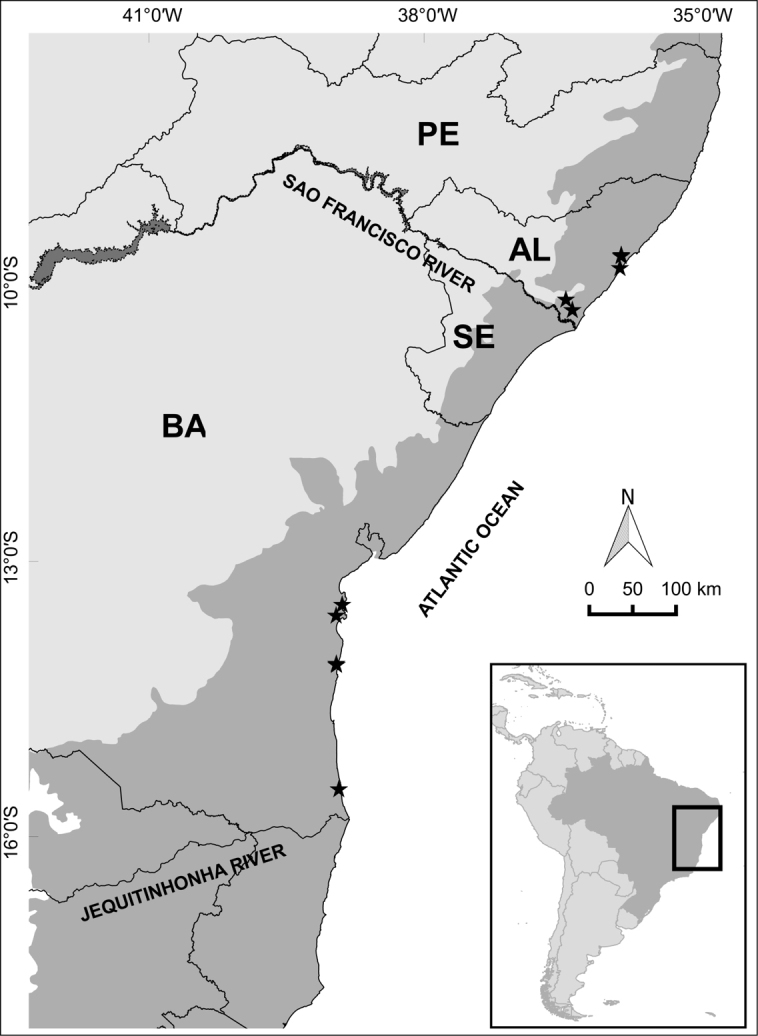
Distribution map of *Eriothecaalversonii*. Gray-shaded areas represent the original extent of Atlantic forest. State boundaries are indicated by continuous lines. Abbreviations for Brazilian states: AL: Alagoas; BA: Bahia; PE: Pernambuco; SE: Sergipe.

## Discussion

*Eriothecaalversonii* is characterized by leaves on fertile branches 1–3-foliolate, petioles up to 8 mm long, leaflets coriaceous, elliptic to broadly-elliptic or narrowly obovate, proximal leaflets up to 27 mm wide, flower buds linear-oblong, and small capsules globose to subglobose up to 21 mm long with glabrous valves. On herbarium sheets, specimens are characterized by terminal vegetative buds with attenuate, falcate apices and by leaves on fertile branches often 1–2-foliolate and often reddish-brown on the abaxial surface.

*Eriothecaalversonii* is morphologically similar to *E.parvifolia* – a shrubby species to 2.5 m tall endemic to the arenitic-quartzite rock outcrops in the Espinhaço Mountain Range in the state of Minas Gerais – by the small petioles, the small, narrowly obovate leaflets with retuse to emarginate apices, and the often persistent leaves on fertile branches; furthermore, both species flower from July to September. However, *E.alversonii* differs from *E.parvifolia* in its oblong-linear (vs. large elliptic) flower buds, calyces 3–4 × 3–5 (vs. 7 × 8–11) mm that are cupuliform and truncate to crenulate (vs. campanulate and mostly 3–5-lobed), stamens ca. 70 (vs. ca. 120), and capsules 15–21 (vs. 30–35) mm long (Table 1).

**Table 1.** Comparison of *Eriothecaalversonii* to morphologically similar species.

Character	*E.alversonii*	*E.macrophylla*	*E.parvifolia*
Leaves on fertile branches	Present	often absent	present
Petiole length of terminal leaf	up to 8	21–45(–65)	8–26(–37)
Number of leaflets of terminal leaf	1–3	3(–5)	(2–)3(–5)
Proximal leaflets width (mm)	8–27	24–66	8–31
Number of secondary veins	7–10	12–18	14–20
Inflorescence position	younger, terminal, often leafy branches	old branches often leafless and modified brachyblasts	terminal, leafy branches
Number of flowers per cyme	1–5	2–7	1–3
Pedicel length (mm)	10–22	14–25	10–15(–25)
Flower bud shape	linear-oblong	broadly elliptic	broadly elliptic
Calyx dimensions (mm)	3–4 × 3–5	5–6 × 7–9	7 × 8–11
Calyx apex	truncate to crenulate	crenulate	mostly 3–5-lobed
Calyx shape	cupuliform	cupuliform	campanulate
Petal dimensions (mm)	15–23 × 3–6	30 × 11	24–31 × 9–15
Number of stamens	ca. 70	140	125
Staminal tube length (mm)	4–5	4	5
Fruit length (mm)	15–21	38–60	27–60
Fruit shape	globose to subglobose	obovoid	obovoid
Seed diameter (mm)	4	ca. 10	6–7
Flowering period	July to September	October to December	July to September
Fruiting period	August to October and December to February	December to February	October to December

*Eriothecaalversonii* emerged as sister to *E.candolleana* (K.Schum.) A.Robyns in a multi-locus DNA sequence-based phylogeny (labeled as ‘*Eriotheca* sp. CS3125’ in Fig. 2B of Carvalho-Sobrinho et al. 2016). It shares with *E.candolleana* the arborescent habit with a regular, well-defined closed crown (“spherical” sensu Ribeiro et al. 1999) and relatively small leaflets and fruit. However, *E.alversonii* can be distinguished readily from *E.candolleana* by 1–3 (vs. 5–9) leaflets that are coriaceous (vs. papyraceous) and glabrous (vs. pubescent with ferrugineous trichomes on the veins abaxially), oblong-linear (vs. globose) flower buds, absence (vs. presence) of glands on the receptacle, and calyces 3–4 × 3–5 (vs. 8–11 × 9–12) mm that are cupuliform (vs. campanulate to tubular), truncate to crenulate (vs. mostly 3–5-lobed), and puberulent to glabrescent (vs. covered with dense ferruginous trichomes). Furthermore, *E.alversonii* inhabits coastal forest over predominantly sandy soils (restinga) in Alagoas and southern Bahia (i.e., 14°S to 10°S) while *E.candolleana* inhabits semideciduous forest on the southeastern Atlantic coast from São Paulo to southern Bahia (i.e., 23°S to 17°S) and in the cerrado biome where it reaches 1,200 m in elevation.

*Eriothecaalversonii* has been frequently misidentified as *E.macrophylla* – a tree species inhabiting restinga and semideciduous forest in the Atlantic coast of northeastern Brazil – probably because in both species the calyces are cupuliform and the leaflets of the fertile branches are 1.5–2.3× longer than wide. However, *E.alversonii* can be readily distinguished from *E.macrophylla* by its terminal, falcate vegetative buds with attenuate apices (vs. straight buds with acute apices), linear-oblong (vs. broadly elliptic to oblong-obovate) flower buds, smaller calyces (3–4 × 3–5 mm vs. 5–6 × 7–9 mm), smaller petals (19–20 × 4–5 vs. 20–32 × 10–15 mm), fewer stamens (ca. 70 vs. 90–140), and smaller fruit (15–21 mm vs. 38–60 mm long) that are globose to subglobose (vs. obovoid). Moreover, *E.alversonii* is characterized by inflorescences borne on younger terminal and often leafy branches while the inflorescences of *E.macrophylla* are borne on old branches that are often leafless and modified as brachyblasts.

## Supplementary Material

XML Treatment for Eriotheca alversonii

## Appendix 1

**Selected herbarium specimens of *Eriothecamacrophylla* and *E.parvifolia* examined for this study.**

*Eriothecamacrophylla* (K.Schum.) A.Robyns

Brazil. **Alagoas**: São José da Lage, BR 104, 11 Nov 2010, *Chagas-Mota 9270* (MAC). **Bahia**: Ilhéus, on road to Vila Brasil, 10 km West of junction with BA001, the junction ca. 40 km south of Ilhéus, just north of the Rio Acuípe, 15°06'S, 39°04'W, 10 May 1993 (lf, fl), *W. Thomas et al. 9843* (CEPEC, NY, SP). Itacaré, entre a Praia do Farol e a Praia da Ribeira, 14 Dec 1992 (lf, fl), *A. Amorim et al. 951* (CEPEC). Itapebi, Faz. Dois Irmãos, Rodovia para Potiraguá, 10 Nov 1970 (lf, fl), *R.S. Pinheiro* & *T.S. Santos* 419 (CEPEC). Jequié, Fazenda Brejo Novo, a 10,5 km da Av. Otávio Mangabeira entrado pela Exupério Miranda no Bairro do Mandacaru, 13°56'53.6"S, 40°06'42"W, 716 m a.s.l., 08 Dec 2004 (st), *G.E.L. Macedo & J.L. Paixão 1499* (HUEFS). Morro do Chapéu, na rodovia, ca. 20 km antes da cidade, 21 Jan 2012 (lf, fr), *J.G. Carvalho-Sobrinho et al. 3305* (HUEFS). Porto Seguro, parte sul entre os municípios de Ajuda e Porto Seguro, 08 Nov 1963 (lf, buds), *A.P. Duarte 7999* (RB, SP). Santa Cruz de Cabrália, Res. Bio. Pau-Brasil, 11 Dec 1971 (lf, fl), *A. Eupunino 94* (CEPEC). Santa Terezinha, 14,5 km na rodovia Elísio Medrado/Sta. Teresinha, Torre da Embratel, ca. 7 km distante do Distr. de Pedra Branca, Serra da Jiboia, 12°51'13"S, 39°28'33"W, 750 m a.s.l., 24 Feb 2000 (lf, fr), *J.G. Jardim et al. 2808* (HUEFS, NY, SPF). **Espírito Santo**: Conceição da Barra, Área 157 da Aracruz Celulose S.A., 28 Oct 1993 (lf, buds) *O.J. Pereira et al. 5163* (VIES). Linhares, Reserva Natural da CVRD, estrada Flamengo, km 08, 03 Dec 2004 (lf, buds), *D.A. Folli 4999* (CVRD); *ibidem*, Reserva Natural da CVRD, estrada Flamengo, km 07, próximo ao pátio, 14 Jan 1994 (lf, fr), *D.A. Folli 2170* (CVRD). **Pernambuco**: Brejo da Madre de Deus, Mata do Bituri, Serra do Prata, próximo do mirante, 08°12'27"S, 36°23'32"W, 920–1030 m a.s.l., *L.M. Nascimento s.n.* (HUEFS 57136, PEUFR).

*Eriothecaparvifolia* (Mart.) A.Robyns

Brazil. **Minas Gerais**: Grão Mogol, estrada para o Rio Ventania, ca. 16°32'S, 42°49'W, 900 m a.s.l., 05 Sept 1990 (lf, fl), *G.L. Esteves CFCR 13348* (HUEFS, SPF); *ibidem*, Serra do Espinhaço, área N Diamantina, campo rupestre near Córrego Lajeado and the junction of the side road from Cristais with the Road Diamantina-Biribiri, ca. 9km NNW-N Diamantina, 1080 m a.s.l., 18°10'S, 43°37'W, *G.L. Esteves CFCR 15511* (NY, SPF); *ibidem*, estrada para Josenópolis, 17 Jul 1998, *G. Hatschbach et al. 67961* (MBM); *ibidem*, Vale do Rio das Mortes, a oeste da cidade, 24 Jul 1986 (lf, fl), *D.C. Zappi et al. CFCR 9892* (BHCB, MAC, R, SPF). Monte Azul, estrada de terra para Formosa, 15°12'S, 42°48'W, 1192 m a.s.l., 27 Oct 2010 (lf, fr), *J.G. Carvalho-Sobrinho et al. 2870* (HUEFS).
